# Impact of antenatal corticosteroid therapy on neonatal respiratory outcomes in late preterm births

**DOI:** 10.1186/s12887-025-05925-w

**Published:** 2025-08-04

**Authors:** Ahmet Kurt, Can Ozan Ulusoy, Dilara Sarikaya Kurt, Sadullah Özkan, Murat Levent Dereli, Aykut Kından, Özge Yücel Çelik, Safiye Elif Uzlu, Şevki Çelen

**Affiliations:** 1 Department of Obstetrics and Gynecology, Ankara Etlik City Hospital, Ankara, Turkey; 2 Department of Perinatology, Etlik City Hospital, Ankara, Turkey; 3 Department of Perinatology, Etlik Zübeyde Hanım Women’s Health Training and Research Hospital, Ankara, Turkey; 4 Department of Neonatology, Etlik Zübeyde Hanım Women’s Health Training and Research Hospital, Ankara, Turkey

**Keywords:** Antenatal corticosteroids, Late preterm birth, Neonatal respiratory outcomes, Cesarean delivery

## Abstract

**Objective:**

This study aimed to evaluate the impact of antenatal corticosteroid therapy (ACT) on neonatal respiratory outcomes in late preterm births, focusing on the interaction between gestational age, mode of delivery, and ACT administration.

**Methods:**

A retrospective case-control study was conducted on 452 singleton late preterm pregnancies (34 + 0 to 36 + 6 weeks) between 2014 and 2021. Among these, 197 patients received ACT, while 255 did not. Maternal and neonatal characteristics were collected, and logistic regression analyses were performed to identify predictors of neonatal respiratory complications.

**Results:**

ACT was associated with a 42% reduction in the odds of neonatal pulmonary complications (adjusted OR: 0.586; 95% CI: 0.347–0.988; *p* = 0.045). Gestational age was a significant protective factor, with each additional week reducing the risk of adverse pulmonary outcomes by 60% (adjusted OR: 0.397; 95% CI: 0.281–0.561; *p* < 0.001). Cesarean delivery was associated with a nearly fourfold increase in pulmonary complications compared to vaginal delivery (adjusted OR: 3.775; 95% CI: 1.859–7.666; *p* < 0.001). ACT showed the most significant protective effect in earlier gestational weeks and among cesarean deliveries.

**Conclusion:**

ACT was associated with reduced neonatal pulmonary complications in late preterm pregnancies, particularly among subgroups with earlier gestational age or cesarean delivery, as supported by multivariate and interaction analyses. These findings underscore the importance of individualized antenatal and perinatal management strategies to optimize neonatal outcomes.

## Introduction

Worldwide, 7.6 million children die before the age of five every year, with premature birth and its complications being the second most common cause of death (14.1%; 1.078 million) [1]. In addition, an estimated 14.9 million pregnancies are complicated by premature births every year. This accounts for 1–11% of all live births worldwide, with rates ranging from around 5% in some European countries to 18% in some African countries [2]. Preterm births are further categorized based on gestational age at birth. Births that occur between 34 + 0 and 36 + 6 weeks gestation are defined as late preterm births and account for approximately 70–75% of all preterm births [3, 4].

Respiratory complications associated with preterm birth, such as respiratory distress syndrome (RDS), the need for respiratory support, necrotizing enterocolitis and intraventricular hemorrhage, are among the greatest challenges for these newborns. The use of antenatal corticosteroids before 34 + 0 weeks gestation is an established universal antenatal intervention aimed at reducing respiratory complications and neonatal mortality [5, 6]. However, the use of ACT between 34 + 0 and 36 + 6 weeks gestation remains controversial. Based on the results of the ALPS (Antenatal Late Preterm Steroid) study, the American College of Obstetricians and Gynecologists (ACOG) recommends a single dose of betamethasone for pregnant women with a delivery risk between 34 + 0 and 36 + 6 weeks’ gestation [5, 7]. However, other randomized controlled trials (RCTs) have shown that the administration of ACT in late preterm delivery does not lead to a significant reduction in neonatal respiratory distress or mortality [8, 9].

The aim of this study is to investigate the effects of antenatal corticosteroid treatment (ACT) on neonatal respiratory outcomes in late preterm infants. By comparing the neonatal outcomes of fetuses that have received ACT with those that have not, this study aims to analyze the interactions between ACT, gestational age and mode of delivery to provide robust evidence for clinical practice. In particular, it aims to determine whether ACT can reduce the increased risks associated with early gestational age and cesarean delivery. These findings should help to optimize perinatal management strategies for late preterm births and thus improve neonatal outcomes in this vulnerable population.

## Materials and methods

This study was conducted as a retrospective case-control study at Etlik Zübeyde Hanım Women’s Health Training and Research Hospital between January 2014 and January 2021. Ethical approval was obtained from the institutional ethics committee with protocol number 01/2021-01-19. The study was conducted in accordance with the universal ethical standards of the Declaration of Helsinki. The clinical trial number is not applicable.

The study included singleton late preterm pregnancies delivering between 34 and 37 weeks of gestation, with and without antenatal corticosteroid treatment (ACT). A total of 452 patients were enrolled, consisting of 197 patients who received ACT and 255 who did not. ACT was administered as a full course of 12 mg betamethasone, given as 6 mg intramuscular injections every 24 h for two doses. All deliveries occurred within 14 days of completing the full course of betamethasone. All patients included in the ACT group were confirmed to have no prior exposure to antenatal corticosteroids. No misclassification was identified during data extraction, as ACT exposure status was verified through detailed chart review and medication administration records. The timing of administration was consistent across the ACT cohort, with all patients receiving a full course within 14 days prior to delivery.

Patients with non-reassuring fetal heart rate patterns at admission, cervical cerclage, congenital anomalies, chromosomal abnormalities, or multifetal pregnancies were excluded. Maternal and neonatal data were obtained from the hospital’s medical record system:

- Maternal Characteristics: Maternal age, body mass index (BMI), history of preterm birth, prior cesarean delivery, hypertensive disorders, diabetes, preterm premature rupture of membranes (PPROM), preterm labor, intrauterine growth restriction (IUGR), oligohydramnios, mode of delivery (cesarean or vaginal), and other obstetric history.
- Neonatal Characteristics: 1- and 5-minute APGAR scores, composite respiratory morbidity (defined as respiratory distress syndrome [RDS], mechanical ventilation, or oxygen requirement), isolated RDS, CPAP or mechanical ventilation use, neonatal intensive care unit (NICU) admission, gestational age, birth weight, and related outcomes.
  Respiratory distress syndrome (RDS) was diagnosed based on clinical symptoms (tachypnea, nasal flaring, grunting, cyanosis) and radiological findings (ground-glass opacities, air bronchograms). Continuous positive airway pressure (CPAP) use was defined as respiratory support with positive airway pressure provided through nasal prongs for at least 6 h. Mechanical ventilation was defined as the need for invasive ventilation via endotracheal intubation for more than 24 h.
- Definition of Composite Pulmonary Outcome: The composite outcome for adverse neonatal pulmonary morbidity was defined as the presence of any one of the following: (1) clinically and radiologically diagnosed respiratory distress syndrome (RDS), (2) requirement of CPAP for at least 6 h, or (3) requirement of mechanical ventilation for more than 24 h. To avoid double counting, each neonate was counted once. If a neonate experienced multiple outcomes (e.g., RDS and mechanical ventilation), they were only counted under the highest level of respiratory support received, establishing a hierarchy as follows: mechanical ventilation > CPAP > isolated RDS.

### Statistical analysis

Normality of continuous variables was assessed using the Kolmogorov-Smirnov test. Continuous variables following a normal distribution were analyzed using the Student’s t-test, while those not normally distributed were compared using the Mann-Whitney U test. Categorical variables were analyzed using the chi-square test. Continuous variables are presented as median values with interquartile ranges (25th and 75th percentiles), while categorical variables are presented as frequencies and percentages (n, %). Variables of clinical importance and relevance to the study aim were further analyzed using univariate and multivariate logistic regression models. A p-value of < 0.05 was considered statistically significant. No missing data were present in the final dataset used for analysis. All included cases had complete records for all relevant variables.

## Results

Demographic, ultrasonographic, obstetric and neonatal findings are compared in Table 1. A total of 452 fetuses were included in the study, with 197 in the ACT group and 255 in the control group. The demographic characteristics, including maternal age, parity, and body mass index (BMI) at booking, were similar between the two groups. However, nulliparity was significantly more common in the control group compared to the ACT group (18.0% vs. 10.7%, *p* = 0.029). The prevalence of hypertensive disorders of pregnancy was significantly higher in the ACT group (14.2% vs. 5.5%, *p* = 0.002).

**Table 1:** Comparison of demographic, ultrasonographic and neonatal outcomes in late preterm fetuses with and without antenatal corticosteroid treatment

	AC Treatment Group (*n* = 197)	Controls (*n* = 255)	*P* value
Demographics			
Maternal age in years, median (25th, 75th percentile)	29.0 (25.0, 33.0)	29.0 (25.0, 34.0)	0.673
Parity, median (25th, 75th percentile)	2.0 (1.0, 2.0)	1.0 (1.0, 2.0)	0.109
Nulliparity, *n* (%)	21 (10.7)	46 (18.0)	***0.029***
Previous Preterm Birth, *n* (%)	75 (38.1)	88 (34.5)	0.434
Maternal body mass index at booking in Kg/m2, median (25th, 75th percentile)	28.0 (25.0, 33.0)	28.0 (26.0, 32.0)	0.803
Gestational diabetes, *n* (%)	1 (0.5)	2 (0.8)	0.719
Hypertensive disorders of pregnancy, *n* (%)	28 (14.2)	14 (5.5)	***0.002***
PPROM, *n* (%)	56 (28.4)	77 (30.2)	0.682
Ultrasonographic Findings			
Estimated fetal weight (gr), median (25th, 75th percentile)	2510 (2218, 2830)	2670 (2399, 2910)	***0.001***
Abdominal circumference (mm), median (25th, 75th percentile)	305 (293, 320)	311 (301, 320)	***0.005***
Oligohydramnios, *n* (%)	11 (5.6)	9 (3.5)	0.297
Polyhydramnios, *n* (%)	12 (6.1)	10 (3.9)	0.288
FGR, *n* (%)	17 (8.6)	14 (5.5)	0.190
Delivery and Neonatal Findings			
Mode of Delivery, *n* (%)			0.679
Vaginal Delivery	59 (29.9)	81 (31.8)	
C-Section	138 (70.1)	174 (68.2)	
Gestational age at birth in weeks, median (IQR)	35.1 (34.3, 35.5)	35.4 (34.6, 36.1)	***< 0.001***
Birthweight in grams, median (IQR)	2500 (2220, 2800)	2620 (2338, 2888)	***0.007***
Composite Adverse Perinatal Outcome, *n* (%)	72 (36.5)	86 (34.0)	0.625
Neonatal Hypoglycemia	21 (10.7)	27 (10.6)	0.980

*P < 0.05 was considered statistically significan t*

Composite adverse neonatal outcome was composed of neonates with APGAR scores below 7 at the 1 st and 5th min and neonates hospitalised in neonatal intensive care unit

*AC* Antenatal Corticosteroid, *PPROM* Preterm premature rupture of membranes, *FGR* Fetal growth restriction

In terms of ultrasonographic findings, the estimated fetal weight was significantly lower in the ACT group compared to the control group (median 2510 g vs. 2670 g, *p* = 0.001). Similarly, abdominal circumference measurements were smaller in the ACT group (median 305 mm vs. 311 mm, *p* = 0.005). The rates of oligohydramnios, polyhydramnios, and fetal growth restriction (FGR) were comparable between the two groups, with no statistically significant differences observed.

Regarding delivery and neonatal outcomes, the mode of delivery (vaginal vs. cesarean section) did not differ significantly between the groups (*p* = 0.679). However, the gestational age at birth was slightly earlier in the ACT group (median 35.1 weeks vs. 35.4 weeks, *p* < 0.001). The birthweight was also significantly lower in the ACT group compared to the control group (median 2500 g vs. 2620 g, *p* = 0.007). Composite adverse perinatal outcomes, including neonates with low APGAR scores or neonatal intensive care unit admissions, were similar between the groups (36.5% vs. 34.0%, *p* = 0.625).

Table 2. compares neonatal pulmonary complications between the ACT group and the control group. The overall rate of pulmonary adverse outcomes, which includes respiratory distress syndrome (RDS), continuous positive airway pressure (CPAP) requirement, and mechanical ventilation (MV) requirement, was slightly lower in the ACT group (17.3%) compared to the control group (20.1%), but this difference was not statistically significant (*p* = 0.448).

**Table 2:** Comparison of neonatal pulmonary complications in patients with and without AC treatment

	AC Treatment Group (*n* = 197)	Controls (*n* = 255)	*P* value
Pulmonary Adverse Outcome, *n* (%)	34 (17.3)	51 (20.1)	0.448
RDS, *n* (%)	20 (10.2)	28 (11.0)	0.777
CPAP requirement, *n* (%)	27 (13.7)	38 (14.9)	0.719
MV requirement, *n* (%)	21 (10.7)	28 (11.0)	0.902

Neonatal Adverse Pulmonary Outcome was composed of postnatal Respiratory Distress Syndrome, neonatal Continuous Positive Airway Pressure and fetuses requiring mechanical ventilation. Fetuses who received CPAP and mechanical ventilation support and developed RDS were included in the analysis as single patients

*RDS* Respiratory Distress Syndrome, *CPAP* Continuous Positive Airway Pressure, *MV* Mechanical ventilation

The rate of RDS was 10.2% in the ACT group and 11.0% in the control group (*p* = 0.777), showing no significant difference. Similarly, the need for CPAP was 13.7% in the ACT group and 14.9% in the control group (*p* = 0.719). The requirement for MV was 10.7% in the ACT group compared to 11.0% in the control group (*p* = 0.902).

Table 3 presents the univariate and multivariate logistic regression analyses for factors associated with neonatal adverse pulmonary outcomes. Gestational age in weeks was significantly associated with a reduced risk of adverse pulmonary outcomes in both univariate (OR = 0.412, 95% CI: 0.296–0.574, *p* < 0.001) and multivariate analysis (aOR = 0.397, 95% CI: 0.281–0.561, *p* < 0.001). This indicates that higher gestational age significantly decreases the likelihood of adverse pulmonary outcomes. ACT also showed a protective effect, reducing the odds of pulmonary complications in both univariate (OR = 0.584, 95% CI: 0.350–0.977, *p* = 0.040) and multivariate analysis (aOR = 0.586, 95% CI: 0.347–0.988, *p* = 0.045). The mode of delivery (cesarean section vs. vaginal delivery) was strongly associated with an increased risk of adverse pulmonary outcomes. Cesarean section significantly increased the odds in both univariate (OR = 3.784, 95% CI: 1.866–7.672, *p* < 0.001) and multivariate analysis (aOR = 3.775, 95% CI: 1.859–7.666, *p* < 0.001). Neither the interval between ACT and delivery (less than 7 days or 7–14 days) nor fetal growth restriction (FGR) showed significant associations in the multivariate model (*p* > 0.05).

**Table 3:** Univariate and multivariate logistic regression analysis for neonatal adverse pulmonary outcome

	Univariate LR		Multivariate LR	
	OR (95% CI)	*P* value	aOR (95% CI)	*P* value
Maternal co-morbidity	0.924 (0.414–2.063)	0.847		
Gestational age in weeks	0.412 (0.296–0.574)	**< 0.001**	0.397 (0.281–0.561)	**< 0.001**
AC Treatment*	0.584 (0.350–0.977)	***0.040***	0.586 (0.347–0.988)	***0.045***
The interval between ACT and delivery is less than 7 days*	0.693 (0.397–1.213)	0.199		
The interval between ACT and delivery is 7–14 days*	0.605 (0.352–1.042)	0.070		
Mode of delivery * (C-section vs. Vaginal delivery)	3.784 (1.866–7.672)	***< 0.001***	3.775 (1.859–7.666)	***< 0.001***
Fetal growth restriction*	0.919 (0.328–2.570)	0.872		
Nulliparity*	0.496 (0.215–1.148)	0.102		

*P* < 0.05 was considered statistically significant

Neonatal Adverse Pulmonary Outcome was composed of postnatal Respiratory Distress Syndrome, Neonatal Continuous Positive Airway Pressure and fetuses requiring mechanical ventilation

*OR* Odds Ratio, *aOR* Adjusted Odds Ratio, *CI* Confidence interval

* Adjusted according to gestational week

**Fig. 1:**
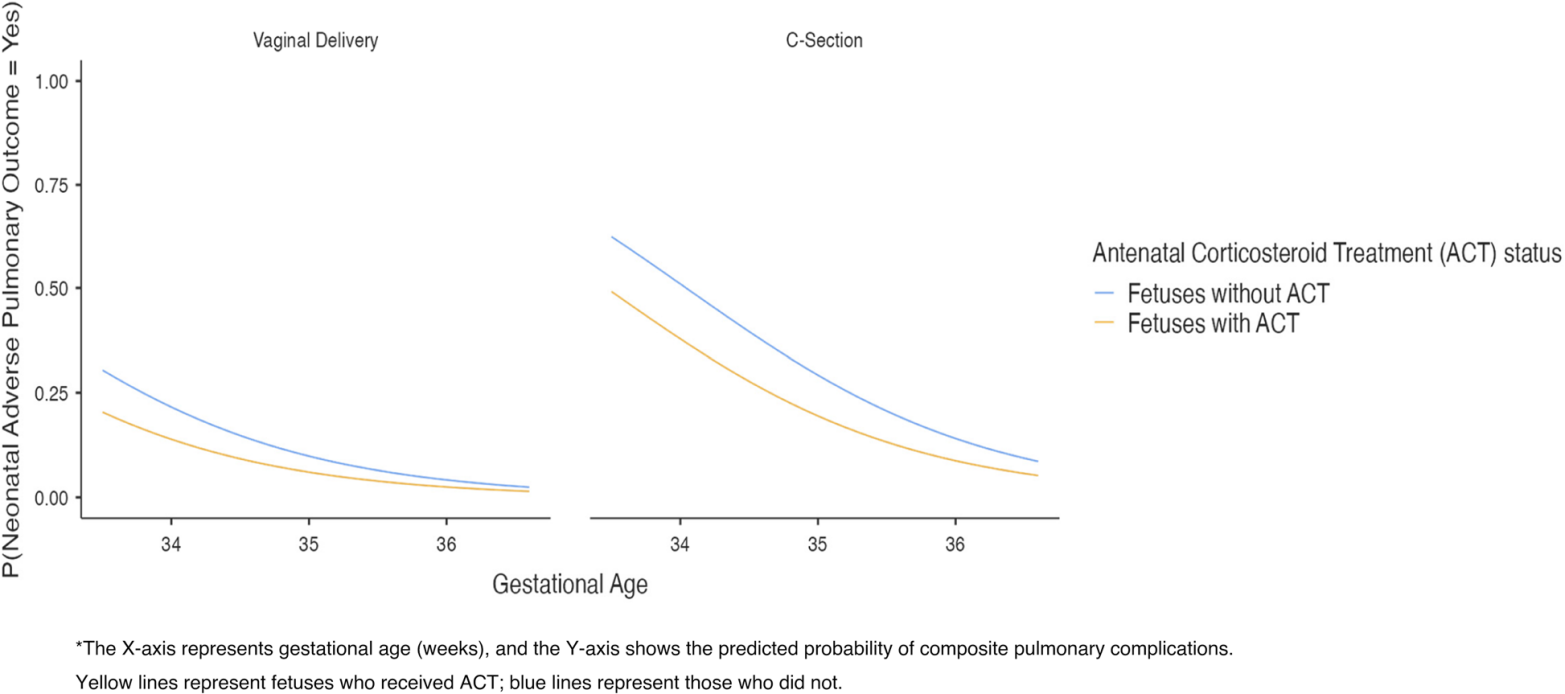
Predicted Probability of Neonatal Adverse Pulmonary Outcomes by Gestational Age, Mode of Delivery, and Antenatal Corticosteroid Treatment (ACT) Status

Figure 1 illustrates the predicted probability of neonatal adverse pulmonary outcomes by gestational age, mode of delivery, and ACT status. The highest probability of adverse outcomes was observed in neonates delivered via cesarean section without ACT at earlier gestational ages. Conversely, the lowest probability was noted in vaginally delivered neonates who received ACT, particularly at later gestational ages. 

Table 4 presents the estimated marginal means of neonatal outcomes based on gestational age, ACT status, and mode of delivery. The worst probability of neonatal pulmonary adverse outcomes was observed in the group of neonates delivered via cesarean section without ACT at 34.4 weeks of gestation, with a probability of 41.3% (95% CI: 31.8–51.4%). This highlights the elevated risk associated with cesarean delivery in the absence of ACT, particularly at earlier gestational ages.

**Table 4:** Estimated marginal means of neonatal outcomes based on gestational age, antenatal corticosteroid treatment (ACT) status, and mode of delivery

Mode of Delivery	Antenatal Corticosteroid Treatment (ACT) status	Gestational Age		Probability	SE	95% Confidence Interval	
						Lower	Upper
Vaginal	Fetuses without ACT	34.4	⁻	0.1570	0.0487	0.0830	0.2769
Delivery		35.2	^µ^	0.0811	0.0262	0.0425	0.1494
		36.0	⁺	0.0402	0.0150	0.0191	0.0824
	Fetuses with ACT	34.4	⁻	0.0983	0.0330	0.0500	0.1843
		35.2	^µ^	0.0492	0.0177	0.0240	0.0980
		36.0	⁺	0.0239	0.0102	0.0103	0.0544
C-Section	Fetuses without ACT	34.4	⁻	0.4128	0.0507	0.3182	0.5143
		35.2	^µ^	0.2500	0.0337	0.1899	0.3215
		36.0	⁺	0.1364	0.0292	0.0885	0.2044
	Fetuses with ACT	34.4	⁻	0.2916	0.0434	0.2143	0.3832
		35.2	^µ^	0.1633	0.0306	0.1117	0.2325
		36.0	⁺	0.0847	0.0242	0.0478	0.1457

⁻ mean − 1SD

^µ^ mean

⁺ mean + 1SD

Conversely, the best probability of neonatal pulmonary outcomes was observed in the group of neonates delivered vaginally with ACT at 36.0 weeks of gestation, with a probability of 2.4% (95% CI: 1.0–5.4%). This emphasizes the protective effect of ACT and the benefit of advancing gestational age, combined with vaginal delivery, in minimizing pulmonary complications.

## Discussion

Our results suggest a statistically significant protective effect of ACT, particularly in certain clinical subgroups. ACT, when adapted to gestational age, is effective in reducing neonatal pulmonary complications. Although the overall rates of RDS, CPAP use, and mechanical ventilation did not significantly differ between groups (Table 2), multivariate regression and interaction models (Fig. 1; Table 4) suggest that ACT may provide greater benefit in specific subgroups, particularly in earlier gestational weeks and cesarean deliveries.The discrepancy between the unadjusted group comparisons (Table 2) and adjusted logistic regression results (Table 3) highlights the importance of accounting for clinical confounders. Once these variables were controlled for in the multivariate model, the independent protective association of ACT became statistically significant. This highlights the importance of evaluating ACT effects within clinical context, rather than assuming a uniform benefit across all late preterm deliveries.

The discrepancy between the unadjusted group comparisons (Table 2) and adjusted logistic regression results (Table 3) highlights the importance of accounting for clinical confounders. In our study, gestational age at birth and mode of delivery had strong associations with neonatal pulmonary outcomes and were not evenly distributed between groups. For example, the ACT group had a slightly earlier gestational age, which may have masked the benefits of ACT in unadjusted analyses. Once these variables were controlled for in the multivariate model, the independent protective association of ACT became statistically significant. This underscores the value of adjusted analyses in retrospective observational studies, where treatment allocation is not randomized.

The protective role of ACT is consistent with the results of the meta-analysis by Saccone and Berghella. This meta-analysis evaluated six randomized controlled trials and showed that the risk of RDS was significantly reduced in infants born to mothers who received antenatal corticosteroids at ≥ 34 weeks of age (RR 0.74, 95% CI 0.61–0.91) [10]. The ALPS (Antenatal Late Preterm Steroids) study, which involved 2,831 patients, also showed that pulmonary complications and neonatal deaths within 72 h occurred in 11.6% of the betamethasone group compared to 14.4% in the placebo group (RR 0.80, 95% CI 0.66–0.97, *P* = 0.02) [7]. The ASTECS study (Antenatal Steroids for Term Cesarean Section) also demonstrated the benefits of antenatal betamethasone administered before elective cesarean sections after 37 weeks [11]. However, there are also randomized controlled trials in the literature with contrary results. Yanuberi et al. reported that the administration of betamethasone in the late preterm period does not reduce the need for treatment of neonatal respiratory distress [12]. The RCT by Porto et al.‘ also came to the same conclusion as the study by Yanuberi [8]. Importantly, our study demonstrated that ACT retained a significant protective effect against neonatal respiratory complications even after controlling for gestational age and other confounders in multivariate analysis, reducing the risk by approximately 42% (aOR = 0.586; 95% CI: 0.347–0.988; *p* = 0.045).

Guidelines for ACT for pregnancies 34 + 0 to 36 + 6 weeks vary. The Society for Maternal-Fetal Medicine (SMFM) and the American College of Obstetricians and Gynecologists (ACOG) recommend ACT for high-risk singleton pregnancies when preterm birth is expected within seven days, but with restrictions (e.g., no chorioamnionitis, no prior ACT course, no tocolysis to delay delivery). The National Institute for Health and Care Excellence (NICE) recommends the use of ACT up to 35 + 6 weeks in preterm labor or planned preterm birth, while the Royal College of Obstetricians and Gynecologists (RCOG) supports ACT up to 34 + 6 weeks in high-risk cases. The Society of Obstetricians and Gynecologists of Canada (SOGC) recommends ACT between 34 + 0 and 36 + 6 weeks based on risks and benefits, but excludes patients with diabetes. The World Health Organization (WHO) and the International Federation of Gynecology and Obstetrics (FIGO) caution against general use of ACT after 34 weeks due to potential risks to the newborn (e.g. hypoglycemia) and limited evidence on long-term outcomes [5, 13–18].

In addition to the protective effect of ACT on pulmonary complications in late preterm labor, another critical aspect is the relationship between the mode of delivery and the adverse effects on the neonate. In our study, delivery by cesarean section was associated with a 3.784-fold (95% CI: 1.866–7.672) higher risk of pulmonary complications than vaginal delivery. At 34 + 4 weeks’ gestational age, the rate of adverse pulmonary outcomes in antenatal corticosteroids treated patients was 29% for cesarean delivery compared to 9% for vaginal delivery. For fetuses not treated with ACT, this rate was 41% for cesarean delivery and 15% for vaginal delivery. The probability of unfavorable pulmonary outcomes decreased with increasing gestational age at delivery.

These findings are consistent with secondary analyzes of the ALPS study by Clapp et al. which showed that the absolute risk difference in neonatal respiratory morbidity between those exposed to antenatal steroids and those not exposed to them varied substantially by gestational age and mode of delivery. Focusing only on the relative risk reduction from prenatal steroids may lead to a false perception of benefit. Instead, presenting patient-specific risk estimates with and without treatment may strengthen shared decision making [19]. Based on the results of our study, we agree with Clapp et al. These gradients of predicted risk support the notion of individualized clinical decision-making, as emphasized by Clapp et al. (2024). Rather than relying on a uniform treatment strategy, clinicians may use such visual probability tools to stratify patients by risk and guide shared decision-making processes. This approach aligns with current SMFM and ACOG guidelines, which advocate for selective use of ACT in late preterm pregnancies based on specific clinical contexts.

Figure 1 in our study visually illustrates the interaction between gestational age, mode of delivery, and ACT status, showing the highest risk in cesarean deliveries without ACT at earlier gestational ages, and the lowest risk in vaginal deliveries with ACT at later gestational weeks. These gradients of predicted risk support the notion of individualized clinical decision-making, as emphasized by Clapp et al. (2024). Rather than relying on a uniform treatment strategy, clinicians may use such visual probability tools to stratify patients by risk and guide shared decision-making processes. This approach aligns with current SMFM and ACOG guidelines, which advocate for selective use of ACT in late preterm pregnancies based on specific clinical contexts.

One of the strengths of this study is its relatively large sample size, which allows for comprehensive multivariable analyzes. The evaluation of late preterm births within a well-defined population and the detailed examination of neonatal outcomes also increases the generalizability of the results. Another major strength of our study is its clear and direct clinical applicability. The findings demonstrate that antenatal corticosteroid therapy (ACT) can be effectively personalized according to gestational age and mode of delivery. Clinicians can use these insights to optimize decision-making processes, significantly improving neonatal respiratory outcomes in late preterm births.

However, some limitations must also be considered. The retrospective design of the study may increase the risk of selection bias. Although the multivariable logistic regression analyses accounted for potential confounding factors, residual confounding by unmeasured variables may still influence the results. Additionally, as a single-center retrospective study, the generalizability of our findings to other populations or different healthcare settings might be limited. Nonetheless, our study provides a more detailed perspective on the interactions between ACT, gestational age and mode of delivery, adding valuable insights to the existing literature. Multicenter prospective studies would help confirm the robustness and applicability of these results.

Furthermore, our study evaluated a single course of betamethasone (12 mg, given twice 24 hours apart), which raises the question of whether different administration regimens might alter the observed effectiveness. Although betamethasone is commonly preferred due to its favorable pharmacokinetic profile, it remains unclear whether alternative corticosteroids like dexamethasone could offer different clinical benefits or risks. Moreover, we did not evaluate long-term neonatal neurodevelopmental outcomes. Investigating these outcomes would provide a more comprehensive understanding of the overall benefit-risk profile associated with antenatal corticosteroid use in late preterm infants.”

The results of this study emphasize the importance of prioritizing the administration of antenatal corticosteroids in patients at risk of preterm delivery in late preterm pregnancies. In particular, the administration of ACT in planned cesarean deliveries should be considered to reduce the risk of neonatal pulmonary complications. In addition, safe prolongation of pregnancy and avoidance of elective cesarean sections without medical necessity are important strategies to improve neonatal outcomes.

Although our study focuses on the short-term respiratory outcomes, it is important to acknowledge the potential risks associated with antenatal corticosteroid use, particularly beyond 34 weeks of gestation. Several studies have raised concerns regarding increased rates of neonatal hypoglycemia following ACT administration, as well as possible long-term neurodevelopmental effects, including behavioral and cognitive outcomes [20, 21]. While our dataset does not include long-term follow-up, these concerns underscore the importance of careful patient selection and shared decision-making when considering ACT in late preterm pregnancies.

Future studies should focus on examining the long-term neurodevelopmental outcomes of preterm infants who have received ACT, as this is important for determining the overall benefit-risk profile of this intervention.

## Conclusion

In conclusion, antenatal corticosteroid treatment helps to reduce neonatal pulmonary complications in late preterm infants. However, the increased pulmonary risks associated with cesarean delivery and timing of delivery emphasize the need for careful decision making in late preterm births with ACT. Optimizing the timing of delivery and prenatal interventions can significantly contribute to improving neonatal outcomes in this vulnerable population.

## Data Availability

Due to hospital policies, patient data and study materials cannot be shared. However, the data are available from the corresponding author upon reasonable request.

## Abbreviations

AC: Antenatal corticosteroid
ACOG: American College of Obstetricians and Gynecologists
ACT: Antenatal corticosteroid therapy
ALPS: Antenatal Late Preterm Steroid
APGAR: Appearance, Pulse, Grimace, Activity, Respiration
BMI: Body mass index
CI: Confidence interval
CPAP: Continuous positive airway pressure
FGR: Fetal growth restriction
FIGO: International Federation of Gynecology and Obstetrics
IQR: Interquartile range
IUGR: Intrauterine growth restriction
MV: Mechanical ventilation
NICE: National Institute for Health and Care Excellence
NICU: Neonatal intensive care unit
OR: Odds ratio
PPROM: Preterm premature rupture of membranes
RCT: Randomized controlled trial
RCOG: Royal College of Obstetricians and Gynecologists
RDS: Respiratory distress syndrome
SD: Standard deviation
SMFM: Society for Maternal-Fetal Medicine
SOGC: Society of Obstetricians and Gynecologists of Canada
WHO: World Health Organization
